# Management of malignant cutaneous wounds in oncologic patients

**DOI:** 10.1007/s00520-022-07194-0

**Published:** 2022-06-08

**Authors:** Michela Starace, Miriam Anna Carpanese, Francesca Pampaloni, Emi Dika, Alessandro Pileri, Daniela Rubino, Aurora Alessandrini, Claudio Zamagni, Carlotta Baraldi, Cosimo Misciali, Annalisa Patrizi, Tommaso Bianchi, Zoe Apalla, Bianca Maria Piraccini

**Affiliations:** 1 Dermatology - IRCCS Policlinico Di Sant’Orsola - Department of Experimental, Diagnostic and Specialty Medicine (DIMES), Alma Mater Studiorum University of Bologna, Bologna, Italy; 2 Dermatology Unit, Department of Medicine, University of Padova, Via Gallucci 4, 35121 Padua, Italy; 3 Addarii Medical Oncology Unit, IRCCS Azienda Ospedaliero-Universitaria Di Bologna, Via Albertoni 15, Bologna, Italy; 4 Second Dermatology Department, Papageorgiou General Hospital, Aristotle University of Thessaloniki, Thessaloniki, Greece

**Keywords:** Neoplastic wounds, Breast cancer, Oncology, Malignant wound, Management of wounds

## Abstract

**Purpose:**

Neoplastic wounds may develop as a result of primary tumor growth in the skin, due to metastasis, or due to skin invasion by tumors emerging from deeper levels. Malignant wounds may present as a crater-like ulcer, or as raised nodules with a cauliflower-like appearance. They are associated with malodor, necrosis, pain, bleeding, and secondary infection. The aim of our study is to better characterize fungating wounds and their management.

**Methods:**

We retrospectively reviewed the database of the Wound Care Unit of the University of Bologna in order to identify individuals affected by neoplastic wound, between January 2019 and February 2021.

**Results:**

We identified 9 females and 2 males with a mean age of 63 years; all were referred by the Oncology Unit. Management differed depending on the characteristics of the patients and the ulcers. Complete healing of the wound, following the parallel complete remission of the lymphoproliferative neoplasia, was observed in one individual. Among the others, one died because of breast cancer, while cutaneous lesions in 2 individuals deteriorated after 1 year of follow-up. Remission/relapse of the ulcer following the treatment course administered for the lymphoma were observed in one patient.

**Conclusions:**

Treatment of malignant fungating wounds is challenging. Considering the neoplastic nature of the wounds, complete healing or improvement cannot be expected with the application of classically prescribed dressing for wounds. A mostly palliative treatment, focusing on maintaining the patient’s quality of life, is a reasonable choice.

## Introduction

A fungating malignant wound is a devastating complication of cancer. It occurs when tumor cells invade the skin and destroy surrounding tissues, infiltrating its supportive vasculature. Neoplastic wounds may result from the growth of a primary skin tumor, from a skin metastasis or from the invasion of the skin by tumors emerging from deeper levels. Local invasion may initially manifest as an inflammation with redness, induration, heat, and tenderness [1]. Malignant wounds may present as either a crater-like ulcer (destructive process) or as raised nodules similar in appearance to a cauliflower (proliferating) or as a combination of both. This justifies the term “fungating,” which is sometimes used to describe proliferating wounds [2–4]. Malignant wounds are characterized by rapid growth and are often associated with malodor, exudate, edema, necrosis, pain, bleeding, pruritus, and infection [3, 5]. Edema, exudate, and necrosis arise from cellular perfusion alteration. Necrotic tissue is a perfect environment for bacterial growth leading to secondary infection. The bacteria that colonize the wound activate proteases that fragment the necrotic tissue, causing the dead tissue to liquefy and generate exudate. The bleeding is the result of an imbalance in hemostatic process. The rapid growth of a tumor can lead to the compression of contiguous structures, such as soft tissues and nerves, causing pain and mobility reduction.

The diagnosis of a malignant ulcer is already suspected on the basis of neoplastic history and clinical presentation, but definite diagnosis demands a skin biopsy and histopathological examination [3].

Malignant fungating wounds affect 5 to 10% of patients with cancer; they usually appear during the last 6 to 12 months of patient’s life but they can be present for years. Wounds of neoplastic etiology can arise from any type of tumor and are more frequently located on the breast, followed by the neck, chest, extremities, genitals, head, and other sites [5, 6].

Patients’ care suffering from malignant fungating wounds requires collaboration of different healthcare specialties, ranging from oncology and palliative care to wound care units [7, 8].

Comprehensive assessment is fundamental in complicated scenarios, such as malignant wounds. The latter may present with unique or atypical clinical presentation and they are always difficult to treat, resulting in dramatical impact on the patients’ and their families’ as well as caregivers’ quality of life [9].

Holistic evaluation of the patients’ physical and emotional profile is essential and should include all aspects of illness, how do they cope with it, and which is the cumulative impact on their lives and families. Furthermore, the psychosocial status, social support, nutritional status, ability to self-care, past and current health morbidities, allergies, coping abilities, and cultural needs should be considered [2, 10, 11]. In regard to the wound assessment, there are various tools that may be applied for the baseline measurements, namely type and depth of wound, presence of necrosis, slough, exudate, malodor, bleeding, pain, condition of the surrounding skin, and signs of infection [10, 12]. Since there is no cure for malignant fungating wounds, treatment is challenging and mainly palliative; it mostly aims to symptoms’ relief, preservation of comfort, and maintenance of patients’ quality of life [7].

Dressing products designed to heal classic wounds of other etiology may not be effective in fungating, ulcers of neoplastic origin. The dressings must be characterized by an appropriate size, thickness, and adhesive capacity; the aforementioned factors, in accordance with the patient preference, will influence the choice of prescription [10, 12]. Although, the literature on the management of malignant wounds is constantly increasing, it is still fragmentary and mostly based on case studies, anecdotal reports, and expert opinions.

Considering the lack of data in the field of neoplastic wounds and their management, we decided to report our real-life experience from a single referral center.

## Materials and methods

**Fig. 1:**
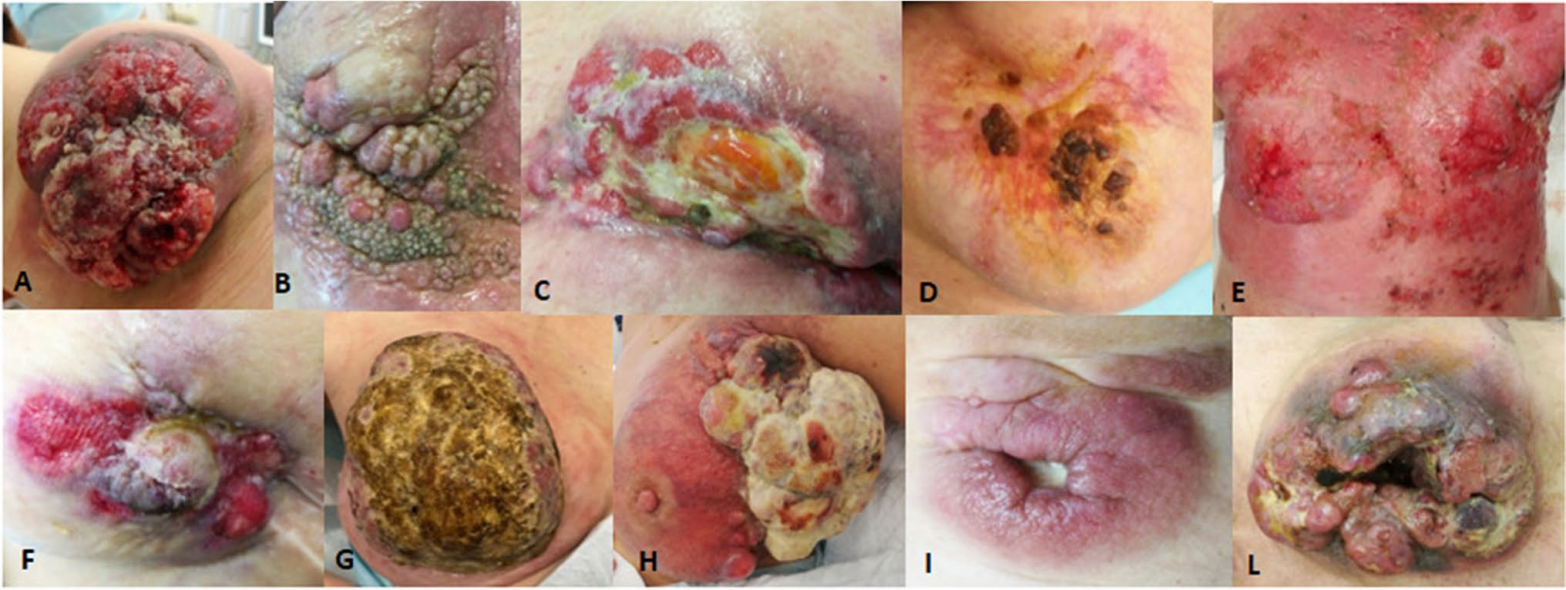
Clinical presentation of our case series: see Table 1 for patient’s characteristics (from “A” to “L”)

A retrospective review of medical records of individuals referred for wounds in the Wound Care Unit at the Dermatology Clinic of University Hospital of Bologna between January 2019 and February 2021 was performed in order to identify patients with histologically diagnosed neoplastic wounds. After written informed consent, we analyzed the charts of attending individuals. We selected 11 patients affected by metastatic cancer who had received the diagnosis of neoplastic wound. Patients were selected in reverse chronologic order, and they had to meet several criteria in order to be included in this evaluation. The inclusion criteria were neoplastic wound histologically confirmed, compliance with treatment regimen, complete medical records, and photographic documentation. The exclusion criteria were previous radiotherapy and malignant degeneration in chronic wound. We collected data regarding demographic information including age, sex, type of cancer, wound size and location, previous oncologic treatments, and onset of the ulcer. As per routine protocol in the Unit, every patient is followed up throughout the therapeutic process, while representative photographs of the initial wound are captured (Fig. 1).

**Table 1:** Data collection of the patients. *M*, male; *F*, female; *Y*, yes; *N*, no

ID	Sex(M/F) Age (years)	Type of cancer and oncologic therapy	Wound size and location	Wound characteristics										Surrounding skin	Dressin**g**
				Exudate	Slough	Granulation	Necrosis	Pain	Malodour	Infection	Bleeding	Depth	Duration (months)		
A	F 61	Metastatic breast cancer Epirubicin Cyclophosphamide	250 cm^2^ Chest	Heavy	Y	N	N	N	N	N	Y	Fungating	2	Erythema	Silver-impregnated foams, polyurethane foam, adsorbent pad
B	F 61	Metastatic breast cancer Ribociclib Anastrozole	250 cm^2^ Chest	Light	N	N	N	N	N	N	N	Fungating	10	Normal	Ozone spray, polyurethane foam
C	F 75	Metastatic breast cancer Vinorelbine Capecitabine Electrochemotherapy with bleomycin	200 cm^2^ Chest	Moderate	Y	Y	Y	Y	N	N	Y	Superficial	2	Erythema	Silver charcoal dressing, adsorbent pad
D	F 38	Metastatic breast cancer Taxol Trastuzumab Pertuzumab	200 cm^2^ Chest	Light	N	Y	N	N	N	N	Y	Fungating	6	Normal	Collagen dressing impregnated with povidone iodine, polyurethane foam
E	F 55	Metastatic breast cancer Not treated at our first evaluation	200 cm^2^ Chest	Light	N	N	N	N	N	N	N	Superficial	12	Erythema	Silver-impregnated foams
F	F 76	Metastatic breast cancer Ribociclib Letrozole Epirubicin Cyclophosphamide	All the chest	Moderate	Y	Y	N	Y	Y	N	Y	Superficial	6	Erythema	Silver charcoal dressing, silver-impregnated foams, polyurethane foam adsorbent pad
G	F 85	Metastatic breast cancer Vinorelbine Trastuzumab Pertuzumab	250 cm^2^ Chest	Heavy	Y	Y	N	N	Y	N	Y	Fungating	8	Erythema	Silver charcoal dressing, silver-impregnated foams, polyurethane foam, adsorbent pad
H	F 55	Metastatic breast cancer Epirubicin Cyclophosphamide Doxorubicin	250 cm^2^ Chest	Moderate	Y	Y	N	Y	Y	N	N	Fungating	5	Erythema	Silver charcoal dressing, polyurethane foam, adsorbent pad
I	F 61	B cell NHL of follicular center cell origin Rituximab + cyclophosphamide + doxorubicin + vincristine + prednisone	200 cm^2^ Leg	Light	Y	N	N	N	Y	Y	N	Crateriform	3	Erythema	Non-adherent dressing impregnated with povidone, silver sulfadiazine cream, polyurethane foam
L	M 71	B cell lymphoma Rituximab Vincristine Loncastuximab Polatuzumab Lenalidomide Pixantrone	250 cm^2^ Chest	Absent	N	N	Y	Y	N	N	N	Fungating	6	Erythema	Silver charcoal dressing, polyurethane foam
M	M 56	Lymphoproliferative chronic syndrome with cutaneous localization Methotrexate Cyclosporin Cyclophosphamide	150 cm^2^ Leg	Absent	N	Y	Y	N	N	N	N	Superficial	18	Normal	Hydrogel dressing

Wound assessment includes the following baseline measurements: type and depth of wound, presence of necrosis, amount of exudate, bleeding, pain, condition of the surrounding skin, presence of malodor, and signs of infection. The diagnosis of a secondary infection is based on the clinical signs (fever, red skin around the ulcer, pain, swelling, purulent exudate), as well as cutaneous swabs and cultures.

We considered the wound characteristics to choose the most appropriate and effective dressing. Available dressings in the Unit are charcoal dressing, silver-based dressing, polyurethane foam dressing, hydro-fiber dressing, alginate dressing, silicon dressing, collagen dressing, non-adherent dressing impregnated with iodopovidone, hydrogel dressing, ozone spray, and absorbent pads.

## Results

We were able to identify 11 patients (9 women and 2 men) affected by metastatic cancer and histologically diagnosed neoplastic ulcer. The average age was 63 years (range 38–85 years). All patients were referred from the Oncology Unit of our Hospital and had a histologic diagnosis of cutaneous metastasis. As shown in Table 1, most of the patients (8/11) were affected by metastatic breast cancer, while three (3/11) had a lymphoproliferative disorder, one had a follicular center, non-Hodgkin B cell lymphoma (NHL), one had a B cell lymphoma, and one had a lymphoproliferative chronic syndrome with cutaneous localization. The TIME framework had been used to aid the assessment of wounds. We observed that patients affected by breast cancer had similar characteristics. More than half of them (5/8) presented with a fungating wound of the breast, while 3 of them had a superficial wound. In seven cases, the wound bed presented with fibrinous slough, with necrosis being present in only one case. All the wounds, regardless of their depth, had a light to heavy exudate. None of them showed signs of infection. All but one patient had already undergone chemotherapy before baseline evaluation. In terms of oncologic therapy, they were treated with different drug protocols. Only one patient had also been treated with electrochemotherapy with bleomycin (see Table 1).

All the 3 patients, affected by a lymphoproliferative disorder, presented with a crateriform ulcer, two on the leg and one on the chest. Two patients did not present any sign of exudate, while one showed a malodorous and purulent exudate and systemic sign of infection (fever); empirical antibiotic therapy was administered (amoxicillin-clavulanate), leading to infection improvement.

Each patient’s wound was cleaned with sodium hypochlorite 0.057 g with active chlorine 0.055 g in 100 mL (Amukine Med®) and then medicated considering the specific characteristics of the wound, the overall health status of the patient, his compliance, and his quality of life.

Heavily or moderately exudative ulcers were managed with adsorbent dressings. Polyurethane foam dressings have a high exudate absorption and a high autolysis debridement capacity. They also avoid odors and keep the skin around the wound intact, and do not cause traumas during removal. Silver-impregnated foams are useful to protect wounds from bacterial colonization, due to the release of ionic silver, and to manage moderate-to-heavy exudate. Hydrogel dressings have also high absorption capacity. They absorb exudates and necrotic residuals by forming a gel with special color and odor characteristics, creating a slightly acidic environment with bacteriostatic properties. Even though only one patient had signs of infection, an antiseptic dressing was used in all individuals to prevent local super-infection; silver charcoal dressings are helpful to control infection and reduce healing times. All the dressings used in our cohort are summarized in Table 1.

**Fig. 2:**
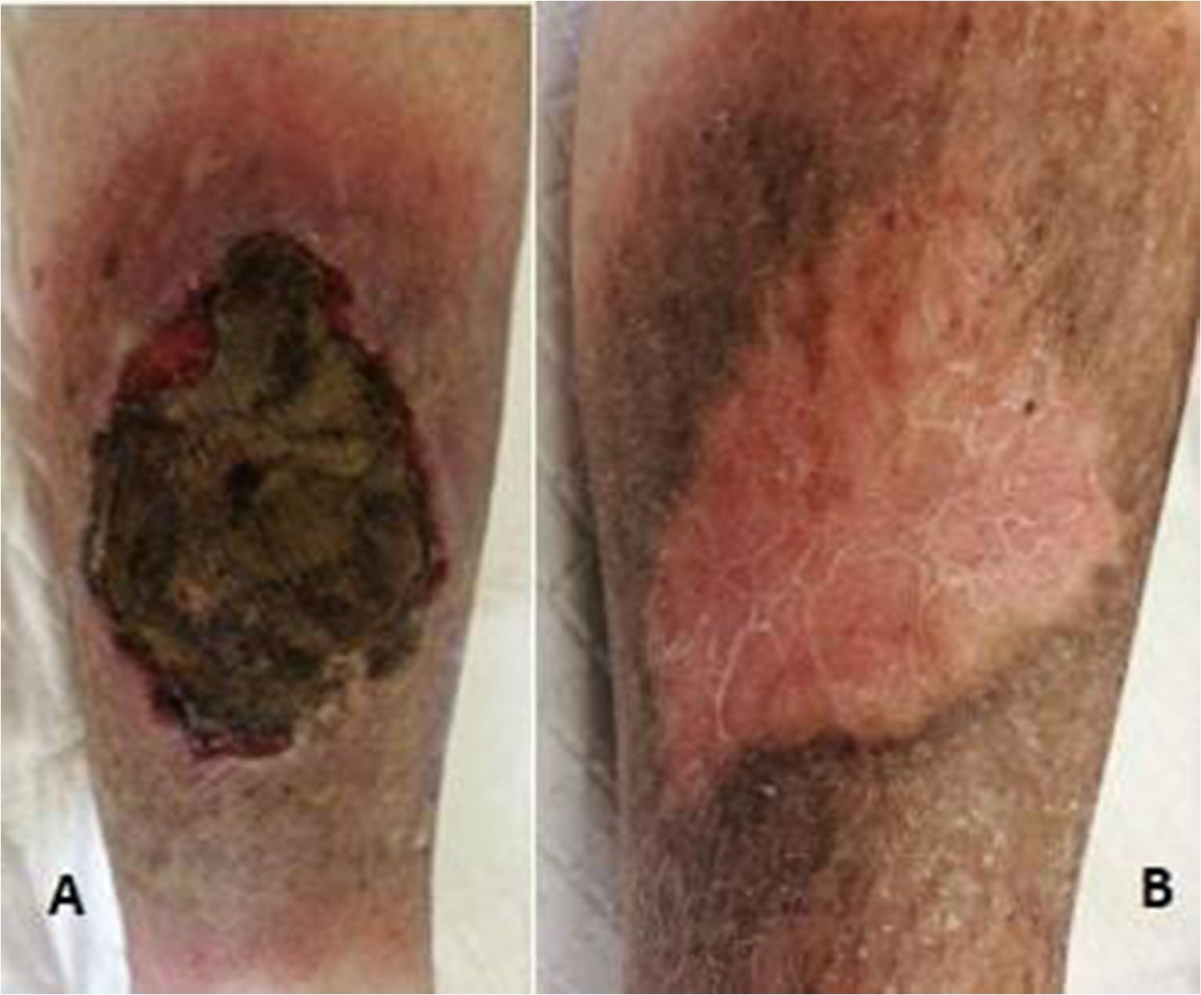
Patient “M.” **A** First clinical evaluation. **B** 6 months post treatment with hydrogel dressing

Complete regression of the ulcer was observed in only one patient affected by a lymphoproliferative tumor (see patient M in Table 1). He discontinued the oncologic treatment due to cancer remission and was treated with hydrogel dressing (see Fig. 2). Among the other patients, one died because of progression of breast cancer; she was treated with silver charcoal dressing, silver-impregnated foams, polyurethane foam, and adsorbent pad; while the others are under ongoing treatment with regular follow-up at the Oncology Unit of the hospital. Two patients affected by breast cancer returned to visit after 1 year from the first consultation. Their tumor had a metastatic progression, and one of them had discontinued the oncologic therapy and started palliative treatment. In both cases, the cutaneous lesion was clinically more fungating and presenting with higher amount of exudate, despite having continued medication with silver charcoal dressing, silver-impregnated foams, polyurethane foam, and adsorbent pad. In one patient affected by lymphoma, we observed subsequent clinical improvement and worsening of the wound depending on the progress of the oncologic treatment prescribed by the hematologist.

After a 3-year follow-up, we could assume that the outcome of our patients was strictly associated with the oncologic prognosis. Despite this, the appropriate management of the wounds can enormously improve the quality of life of patients, always considering the psychosocial factor that influences the care [12–14].

## Discussion

There are many clinical challenges in providing medical care for patients with neoplastic wounds. Since there are no clearly defined wound care guidelines, many centers follow their own protocols to treat this type of wounds [2, 15].

In our outpatient service, we deal with oncologic patients with different and challenging wound management. All individuals were previously evaluated at the Oncology Unit and had a histologic diagnosis of cutaneous metastasis; they had been already treated with different chemotherapy protocols before dermatologic consultation. Only one patient, affected by breast cancer, performed electrochemotherapy with bleomycin, which is a well-known therapeutic option for skin metastases when other local and systemic therapies fail. Larger or ulcerated lesions, especially in previously irradiated areas, seem to have a worse outcome and a higher toxicity. The very large wound size, as well as the presence of ulceration in our sample, did not allow us the application of electrochemotherapy and bleomycin [16, 17].

The TIME (Tissue, Inflammation/infection, Moisture imbalance, Epithelial edge advancement) paradigm is a systematic approach for assessing chronic wounds; it aspires to create an optimal environment establishing a vascularized and stable wound bed. This approach is fundamental because fungating wounds usually do not progress through the normal process of wound healing (hemostasis, inflammation, proliferation, maturation), resulting in challenging chronic wound [18, 19].

Among the symptoms associated with malignant wound, malodor has been often reported as the main cause of distress to patient and their family [20]. The malodor etiology is not well understood; however, it has been proposed that the presence of devitalized tissue provides an excellent environment for bacteria growth (aerobic and anaerobic) leading to bad odor. Another contributing factor could be the saturation of dressing; therefore, their daily change is fundamental. Finally, malodor can occur when an ulcerating wound extends into a body cavity, increasing exudate associated with the wound [21–23]. Patients included in the current case series did not complain about malodor. The use of silver dressings can be very effective to reduce malignant fungating wound malodor [15]. A heavy exudate, sometimes over a liter per day, often complicates malignant wounds. Its management is cumbersome, usually necessitating frequent dressing changes, about two or three times a week, to avoid risk of maceration of the peri-wound skin that may contribute to wound enlargement. Control of exudate in neoplastic wounds is important to reduce also other symptoms like malodor, bleeding, and increased patient comfort [22]. Heavy exudate may result from autolytic or enzymatic debridement; this procedure, although useful, may increase the risk of bleeding. In fungating wounds, autolytic debridement is preferred over mechanical debridement, because it reduces the risk of bleeding. It can be promoted with any dressing regimen that maintains a moist wound surface [11].

Most of our patients, in particular those with breast cancer–associated wounds, presented with moderate-to-heavy exudate. Its management has been one of the most challenging parts of the treatment. We often applied an absorbent dressing, but in many cases this was not enough. In this context, for these individuals, apart from the main dressing, we applied an additional adsorbent pad, teaching patients to change this part of the dressing by themselves.

Another symptom that though rare may pose difficulties in management is bleeding. The low frequency of bleeding is probably due to the lack of data on the hemorrhagic malignant wounds. Local methods to manage bleeding can include special dressing, radiotherapy, and systemic treatments like etamsylate, increasing the endothelial resistance of capillaries and promoting platelet adhesion. Tranexamic acid can be also used but it can be risky to apply for patients affected by thrombogenic paraneoplastic syndrome [15]. Bleeding is associated with a poor prognosis, so an early identification of bleeding in malignant lesions may help to evaluate and start palliative care with the objective to improve the quality of patient life. Many studies showed positive results with topical hemostasis, but scientific evidence is still weak and emerges from studies with poor methodological quality [24, 25]. In our case series, only one patient presented with bleeding and required hemostatic dressing and blood transfusion, as also reported in other studies [25].

Pain is a significant complication of many malignant wounds; application of a management protocol for pain is fundamental and should be always based on a multidisciplinary assessment. There are several types of pain associated with malignant wounds: deep pain, neuropathic pain, and superficial pain related to procedures. Analgesia prior to dressing removal is often mandatory and needs to be given in advance in order to ensure that the analgesic effect will reach its maximum level of action at the desirable time-point [4, 26]. In our cohort, three individuals reported pain and were treated with analgesic therapies. Particular attention during dressing changes, with application of a pre-medication with topical anesthesia and use of non-adherent dressing, is strongly recommended. Every affected patient may experience pain and in this context all of them should be evaluated in a multidisciplinary team [18].

In the end, consideration should be given in the presence of infection. As we already said, malignant wounds are often associated with malodor, exudate, pain, and bleeding and peri-wound erythema that may represent signs of infection. A swab culture and antibiotic susceptibility should be performed in any suspicious of infection wound [12, 27, 28]. In our series, only one patient had an episode of infection. The low rate of infections is probably related to the pre-emptive application of an antiseptic dressing in all of our patients.

The management we followed in our patients differed from the strategies usually described in the literature.

In particular, we always applied an antiseptic dressing, such as silver or iodopovidone impregnated dressings, to prevent infections and malodor. Accordingly, in case of bleeding, a collagenous dressing as hemostatic tool was offered. An absorbent dressing (polyurethane foam, alginate foam, adsorbent pad) was applied in presence of exudate [29–31]. After a diagnosis of neoplastic wound, a close follow-up should be performed. During the first period after diagnosis, we examined patients twice a week.

## Conclusions

Treatment of malignant fungating wounds is challenging. A mostly palliative treatment, focusing on maintaining the patient’s quality of life, is a reasonable choice.

Patients suffering from malignant fungating wounds commonly experience significant physical and psychosocial impact. The disease burden is devastating for them and their family members. A wound care intervention, built on evidence-based practice and psychosocial support, can increase the patient’s sense of well-being and reduce difficult psychosocial problems.

Despite the excellent management of wounds, it must always be considered that these lesions rarely improve or heal. Starting a palliative care if there is a progressive disease and deterioration of the general conditions is a reasonable decision.

## Limitations

Due to the limited sample size, further studies with larger sample size are needed to make definitive recommendations. Also, because every wound is unique, standardizing therapy for a particular etiology is difficult in a limited case series.

## Data Availability

Data available on request from corresponding author.
